# A phylogenomic approach to resolving interrelationships of polyclad flatworms, with implications for life-history evolution

**DOI:** 10.1098/rsos.220939

**Published:** 2023-03-29

**Authors:** Jessica A. Goodheart, Allen G. Collins, Michael P. Cummings, Bernhard Egger, Kate A. Rawlinson

**Affiliations:** ^1^ Division of Invertebrate Zoology, American Museum of Natural History, New York, NY 10024, USA; ^2^ Scripps Institution of Oceanography, University of California, San Diego, La Jolla, CA 92037, USA; ^3^ NMFS, National Systematics Laboratory, National Museum of Natural History, Smithsonian Institution, MRC-153, PO Box 37012, Washington, DC 20013, USA; ^4^ Center for Bioinformatics and Computational Biology, University of Maryland, College Park, MD 20742, USA; ^5^ Universität Innsbruck, Department of Zoology, Technikerstr. 25, 6020 Innsbruck, Austria; ^6^ Wellcome Sanger Institute, Hinxton, Cambridgeshire CB10 1SA, UK; ^7^ Josephine Bay Paul Center, Marine Biological Laboratory, Woods Hole, MA, 02543

**Keywords:** polyclad phylogeny, transcriptomics, flatworms, Polycladida, development, larval evolution

## Abstract

Platyhelminthes (flatworms) are a diverse invertebrate phylum useful for exploring life-history evolution. Within Platyhelminthes, only two clades develop through a larval stage: free-living polyclads and parasitic neodermatans. Neodermatan larvae are considered evolutionarily derived, whereas polyclad larvae are hypothesized to be ancestral due to ciliary band similarities among polyclad and other spiralian larvae. However, larval evolution has been challenging to investigate within polyclads due to low support for deeper phylogenetic relationships. To investigate polyclad life-history evolution, we generated transcriptomic data for 21 species of polyclads to build a well-supported phylogeny for the group. The resulting tree provides strong support for deeper nodes, and we recover a new monophyletic clade of early branching cotyleans. We then used ancestral state reconstructions to investigate ancestral modes of development within Polycladida and more broadly within flatworms. In polyclads, we were unable to reconstruct the ancestral state of deeper nodes with significant support because early branching clades show diverse modes of development. This suggests a complex history of larval evolution in polyclads that likely includes multiple losses and/or multiple gains. However, our ancestral state reconstruction across a previously published platyhelminth phylogeny supports a direct developing prorhynchid/polyclad ancestor, which suggests that a larval stage in the life cycle evolved along the polyclad stem lineage or within polyclads.

## 1. Background

Flatworms (Platyhelminthes Minot, 1876) are among the most diverse invertebrate phyla, with an estimated 100 000 parasitic and free-living species [1]. They belong to the group of animals called Spiralia Schleip, 1929, which includes 12 other phyla; nemerteans, annelids, phoronids, ectoprocts, brachiopods, gastrotrichs, molluscs, entoprocts, chaetognaths, rotifers, micrognathozoans and gnathostomulids [2]. Many spiralians undergo indirect development whereby the embryo develops into the young adult form through a distinct larval stage. There are notable similarities in the structure and functional roles of larval characters among spiralians, such as ciliary bands for swimming and feeding [3], and these have led to hypotheses of their homology [4]. Two clades within flatworms, Polycladida Lang, 1881 and Neodermata Ehlers, 1984, contain taxa with indirect development [5–8]. Although the complex life cycles of neodermatan flatworms and their larvae have been considered evolutionarily derived (i.e. an intercalated larval stage) [9], the biphasic life cycle of polyclads has been considered the ancestral condition for Platyhelminthes [9], and thought to be retained from the last common ancestor of Spiralia due to similarities in larval ciliary bands and spiral cleavage patterns [4]. Recent phylogenomic analyses have, however, produced topologies for Platyhelminthes that call this hypothesis into question [10,11]. The position of polyclads recovered in these analyses suggests that it is more parsimonious to view polyclad larvae as one or more independent acquisitions limited to this group [11].

**Figure 1.**
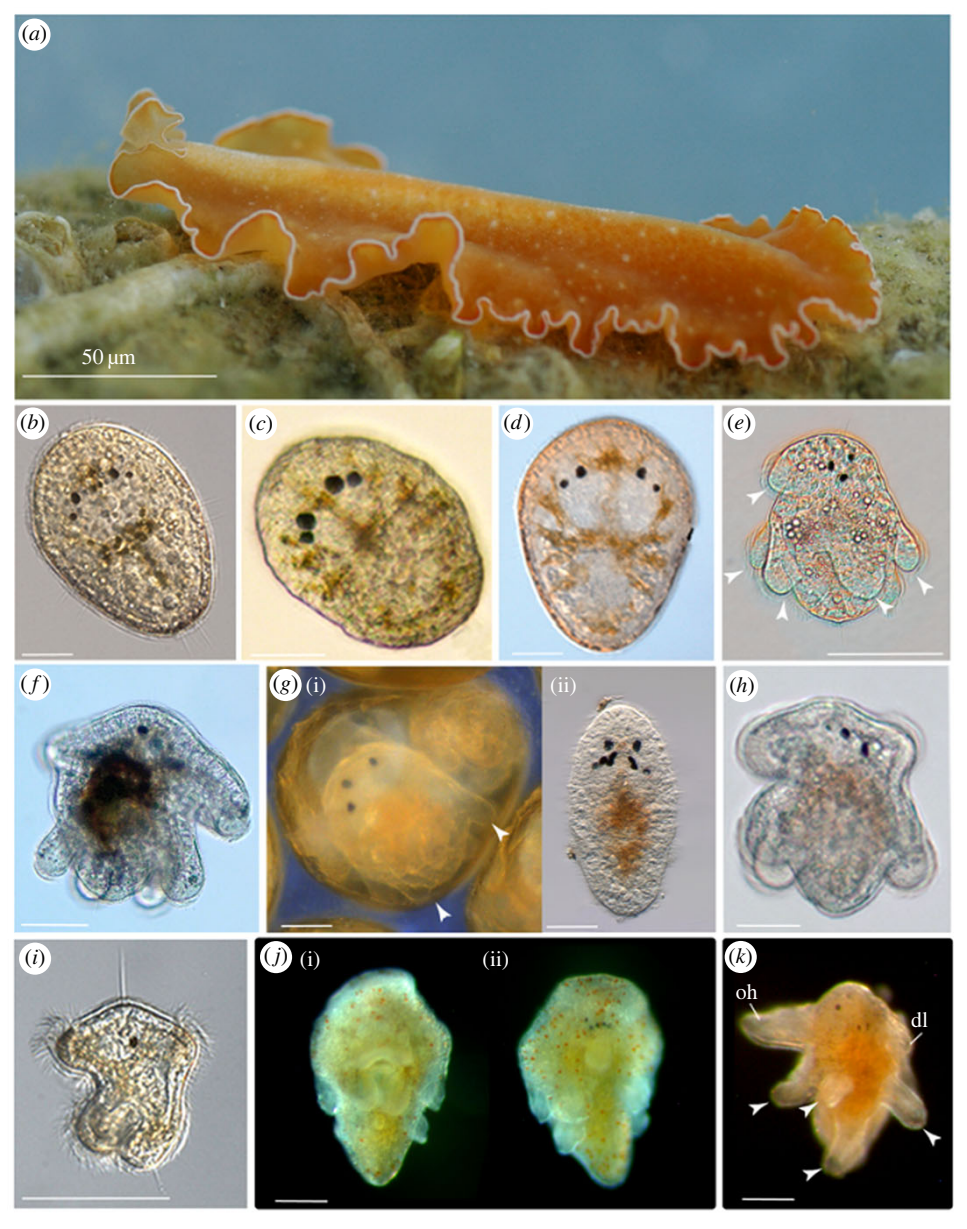
Polyclad flatworms are dorsoventrally flattened as adults, some species develop directly into this body plan, others develop indirectly through larval forms with transient features such as lobes and ciliary tufts and bands. (*a*) Adult polyclad, *Yungia* sp. (*b–d*) Hatchlings of direct developing species; *Euplana gracilis* (*b*), *Notocomplana* sp*.* (Schmarda, 1859) (*c*) and *Echinoplana celerrima* Haswell, 1907 (*d*). (*e–k*) Hatchlings of indirect developing taxa; newly hatched Müller's larva of *Cycloporus gabriellae* Marcus, 1950, showing long motile cilia on lobes (arrowheads) (*e*), newly hatched Müller's larva of *Prosthecereaus crozieri* (Hyman, 1939) (*f*); *Prosthiostomum acroporae* shows ‘intermediate development’ with the embryo developing eight larval lobes (arrowheads) inside the egg capsule (*g* (i)), but most individuals undergo intra-capsular metamorphosis—reabsorbing larval lobes before hatching (*g* (ii)); Müller's larva of *Prosthiostomum siphunculus* (Delle Chiaje, 1822) (*h*), four-lobed Goette's larva of *Stylochus ellipticus* (Girard, 1850) (*i*), ventral (*j* (i)) and dorsal (*j* (ii)) view of 10-lobed larva collected from the plankton, species unknown; and lateral view of 10-lobed larva collected from the plankton (*k*). B-K scale 50 µm. Species in B-H are sequenced in this study. (Images (*i–k*) are from Rawlinson [14].)

Polyclads (order Polycladida) offer an interesting group within which to explore the evolution of life-history strategies and larval characters. They are a clade of marine flatworms that, as adults, are generally found on the seafloor in coastal habitats (figure 1*a*), but they have also been collected in the deep sea and in the water column [12,13]. The most common mode of development in Polycladida might be direct [14], whereby the hatchling resembles a sexually immature adult worm, i.e. dorsoventrally flattened (figure 1*b–d*), with a uniform covering of motile cilia. However, there are many species that develop indirectly, through a planktonic phase that has transient larval features which make it morphologically distinct from the adult form. These larval features include lobes, or protrusions, upon which are bands of longer motile cilia used for swimming in the water column (figure 1*e–k*). These features are lost at metamorphosis and the transition to a benthic niche [8,15]. Polycladida has traditionally been divided into two suborders based on multiple morphological characters of the adult worms, including the presence (Cotylea Lang, 1884, approx. 350 species) or absence (Acotylea Lang, 1884, approx. 450 species) of a ventral adhesive structure [16–18], known as a cotyl [19,20]. Most cotyleans examined to date hatch as an eight-lobed larval stage (known as Müller's larva) (e.g. figure 1*e,f*,*h*). There are four known exceptions; *Prosthiostomum acroporae* (Rawlinson *et al*., 2011) undergoes intra-capsular metamorphosis—reabsorbing its eight larval lobes before hatching (intermediate development) [21] (figure 1*g*), *Pericelis cata* Marcus & Marcus, 1968 exhibits poecilogony (i.e. hatchlings emerge from an egg plate with and without larval characters) [22], *Boninia divae* Marcus & Marcus, 1968 hatchlings have reduced lobes [22], and *Theama mediterranea* Curini-Galletti *et al*., 2008 hatches as juveniles without lobes and ciliary bands [23]. By contrast, taxa assigned to Acotylea mainly exhibit direct development, but species with intermediate and indirect development do exist, with a diversity of larval morphologies (e.g. six- and eight-lobed Müller's larvae, four-lobed Goette's larva (figure 1*i*) and an eight-lobed, dorsoventrally flattened Kato's larva [14]). Larvae with 10 lobes have also been described [9,14,24] (figure 1*j*,*k*).

Recent molecular phylogenies of Polycladida have significantly increased our understanding of the interrelationships within the order [20,25–34]. However, these studies used one or a few genes for their inferences, primarily 18S, 28S and COI. It is well known that phylogenetic inferences using single gene data matrices may represent limited approximations of the relationships among taxa (as they represent gene trees as opposed to species trees) [35] and are subject to limitations in scope due to differences in rates of evolution across genes (e.g. [36]). They also offer a comparatively low number of phylogenetically informative positions. In the case of polyclads, these challenges have led to phylogenies that are often well-supported near the tips but provide lower resolution of earlier branching lineages in the polyclad tree.

The two goals of this paper were to generate a well-supported phylogenetic hypothesis for Polycladida inferred from transcriptomic data, and to use this framework to assess the evolutionary origin of a larval stage (i.e. indirect development) in polyclads. The use of transcriptomes for phylogeny removes the potential bias related to selecting specific genes for analysis *a priori*. We generated RNA-Seq data from 21 polyclad species and developed a data matrix that included a further six transcriptomes for polyclads, and nine transcriptomes for non-polyclad flatworms. We scored the mode of development for these taxa from the literature and personal observation. Here, indirect development was scored if the species has distinct and transient characters that are not retained in the adult body plan (i.e. larval characters, specifically lobes and/or ciliary bands/plates/tufts), whereas direct development was called if no larval characters are recorded during embryogenesis and at hatching. We used the phylogenomic relationships to reconstruct the ancestral mode of development among the polyclad lineages in our tree to discern where development modes originated. Finally, we used a previous phylogenomic-based tree of the phylum to infer the ancestral mode of development at nodes within the flatworm phylogeny. This enabled us to determine whether it would be phylogenetically congruent to consider indirect development (and associated characters, such as larval ciliary bands) homologous among polyclad families and suborders, and among different flatworm orders (i.e. polyclads and neodermatans). This work represents an important step in our comprehension of polyclad phylogeny and life-history evolution among flatworm clades.

## 2. Results

### 2.1. Assembly and data matrix properties

Our final data matrix includes transcriptomes of 27 polyclad species from at least 23 genera (electronic supplementary material, table S1), plus nine outgroup taxa representing other platyhelminth lineages (Catenulida Meixner, 1924, Macrostomorpha Doe, 1986, Gnosonesimida Karling, 1974, Cestoda Gegenbaur, 1859 and Tricladida Lang, 1881) [11]. Once assembled, the number of contiguously assembled sequences (contigs) per sample ranged from 62 613 (*Xenoprorhynchus* sp. I Laumer & Giribet, 2014) to 819 086 (*Prostheceraeus vittatus* (Montagu, 1815)) ($\bar{x}=159\;485$; electronic supplementary material, table S2). N50 ranged from 520 bp (*Xenoprorhynchus* sp. I) to 2041 bp (*Boninia divae*) ($\bar{x}=1430\;\mathrm{bp}$; electronic supplementary material, table S2), and Benchmarking Universal Single-Copy Orthologs (BUSCO) scores (compared to the metazoa_odb10 database) ranged from 28.8% complete (*Xenoprorhynchus* sp. I) to 91.4% complete (*Prosthiostomum siphunculus*) with all new polyclad transcriptomes surpassing 83% complete. Our data matrix consisted of 4469 orthologous groups and 5 081 724 nucleotide positions (58.0% complete with 0.32% ambiguous characters; electronic supplementary material, table S2). Individual species ranged from 848 391 bp (16.7% of full alignment length, *Xenoprorhynchus* sp. I) to 3916323 bp (77.1% of full alignment length, *Leptoplana tremellaris* (Müller OF, 1773)), with an average of 2 947 307 bp across all species (electronic supplementary material, table S2).

### 2.2. Phylogenetic analyses

**Figure 2.**
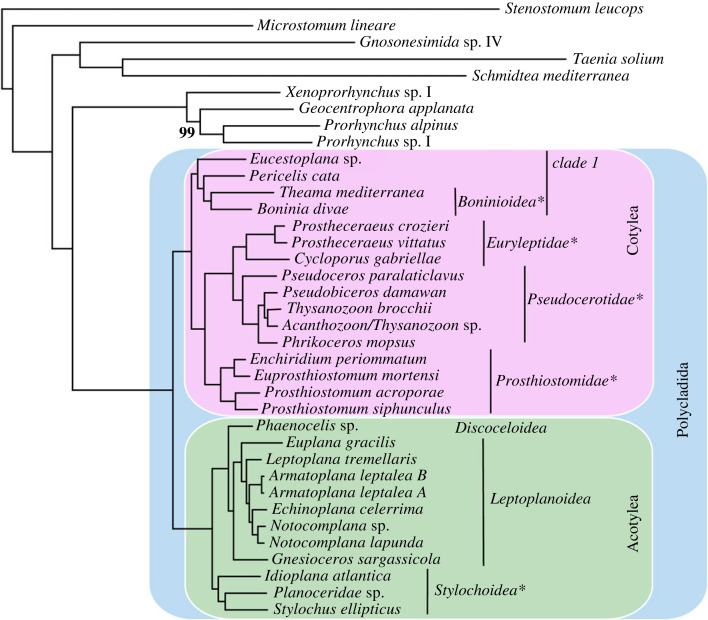
Maximum-likelihood phylogeny of Polycladida constructed using RAxML-NG from a concatenated nucleotide matrix of 4469 genes partitioned by codon position. Unless indicated otherwise, all branches have 100% BS. Each branch also has 100% BS in our IQ-TREE analysis accounting for heterotachy. The blue box indicates Polycladida, and the pink and green boxes indicate the clades for Cotylea and Acotylea, respectively. Superfamily and family-level systematic affinities supported by this analysis are marked with an asterisk.

The inferred topologies were identical across all three analyses (with some variation in branch lengths), with 100% bootstrap support (BS) for all branches in our analysis accounting for heterotachy. Here we report results only for our maximum-likelihood phylogeny of Polycladida constructed using RAxML-NG with our matrix partitioned by codon position (figure 2). Results from the other two analyses are provided in our Dryad repository (https://doi.org/10.6076/D1JG60). All 20 search replicates in our partitioned analysis returned the same best tree topology (figure 2) and [37] indicate strong support for the monophyly of Polycladida (BS = 100) and the taxonomic sub-clades Cotylea *sensu* Bahia *et al*. [27] (BS = 100) and Acotylea (BS = 100) *sensu* Dittmann *et al*. [28]. The enigmatic genera *Cestoplana* Lang, 1884, *Pericelis* Laidlaw, 1902*, Boninia* Bock, 1923, and *Theama* Marcus, 1949 fall within Cotylea, and form a novel clade, clade 1 (BS = 100; figure 2), that is sister to the rest of cotyleans (BS = 100). Within clade 1, Boniniidae Bock, 1923 and Theamatidae Marcus, 1949 are sister taxa, supporting the recently proposed superfamily Boninioidea Bock, 1923 [28]. The cotylean families Prosthiostomidae Lang, 1884, Pseudocerotidae Lang, 1884, and Euryleptidae Stimpson, 1857, are recovered with high support (all with BS = 100).

In Acotylea, there are three superfamilies: Stylochoidea Stimpson, 1857, Leptoplanoidea Ehrenberg, 1831 and Discoceloidea Laidlaw, 1903. Our results are consistent with a monophyletic Stylochoidea *sensu* Dittmann *et al*. [28] (BS = 100), and its position as the sister lineage to the rest of the suborder [20,27,28]. Leptoplanoidea *sensu* Dittmann *et al*. [28] is rendered paraphyletic by the inclusion of *Euplana gracilis* Girard, 1853 (BS = 100) (electronic supplementary material, table S1). As a single undescribed species of *Phaenocelis* Stummer-Traunfels, 1933, is the only confirmed taxon of Discoceloidea included in our analysis, support for this superfamily could not be assessed.

Gnesiocerotidae Marcus & Marcus, 1966 (*sensu* Faubel 1983) represented here by *Gnesioceros* Diesing, 1862 and *Echinoplana* Haswell, 1907, was not supported (electronic supplementary material, table S1). The newly created family Notocomplanidae Litvaitis *et al*. [20] was monophyletic. The families Stylochidae Stimpson, 1857, Idioplanidae Dittmann *et al*. [28], Cryptocelidae Laidlaw, 1903, Euplanidae Marcus & Marcus 1966, are represented by only one species in our analysis, therefore monophyly of these families could not be assessed.

### 2.3. Ancestral character estimation

**Figure 3.**
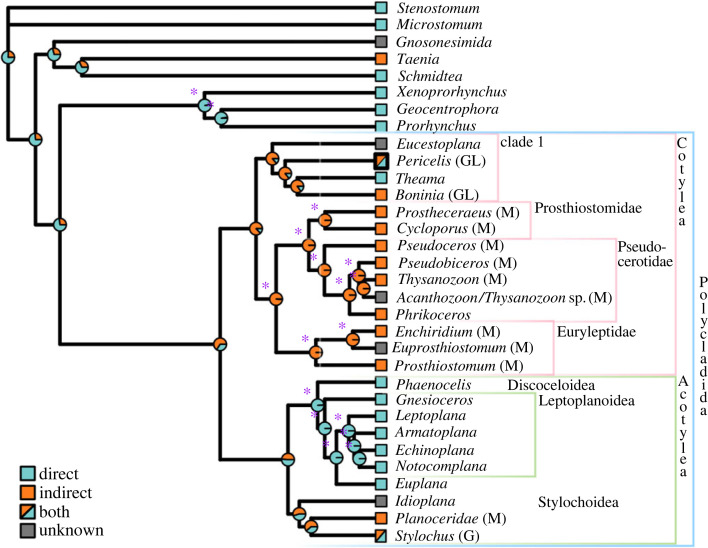
Ancestral state reconstruction analysis in Polycladida for the presence of indirect (orange), direct (blue) development or both strategies in the genus (orange/blue; thin border denotes species within the genus have both strategies; thick border denotes some species within the genus exhibits poecilogony). Pie charts on the nodes are scaled marginal likelihoods calculated using the ace function in APE. The purple asterisks denote significant nodes as defined by proportional likelihood significance tests [38] with a likelihood difference of at least 2 or higher. Type of larva in brackets after genus name; M = Müller's, G = Goette's and GL = Goette's-like.

**Figure 4.**
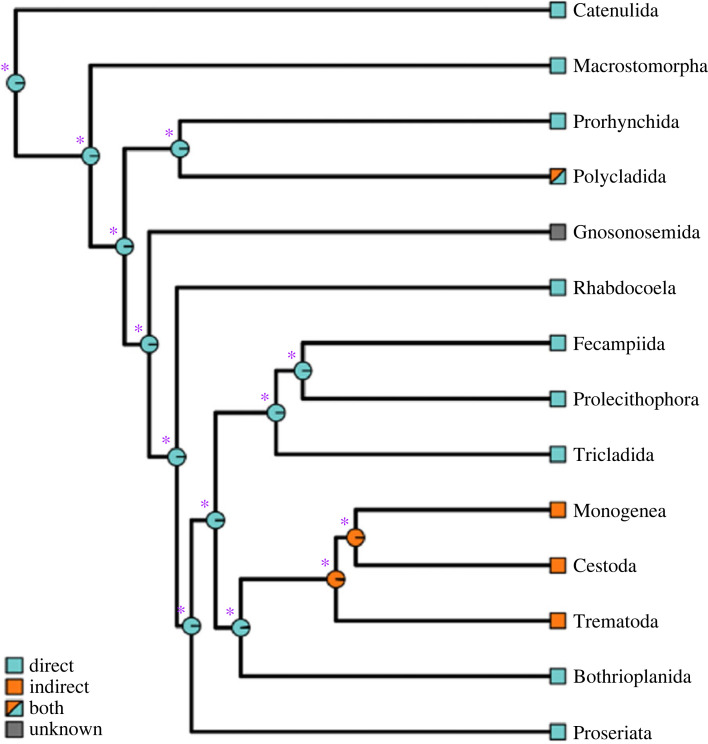
Ancestral state reconstruction analysis in Platyhelminthes for the presence of indirect (orange), direct (blue) development or both strategies in the order (orange/blue). Pie charts on the nodes are scaled marginal likelihoods calculated using the ace function in APE. The purple asterisks denote significant nodes as defined by proportional likelihood significance tests [38] with a likelihood difference of at least 2 or higher.

Mode of development is unknown for 11 of the 36 species (31%) in our phylogeny (electronic supplementary material, table S3). However, for 6 of these 11 species, there is developmental information for one or more congeneric species. Therefore, we coded the mode of development at the genus level for all taxa in our inferred polyclad phylogeny (except an undescribed Planoceridae species that we coded at the family level). Our ancestral state reconstruction shows indirect development at the base of a clade of cotyleans that includes the families Prosthiostomidae, Euryleptidae and Pseudocerotidae; direct development at the base of a clade of acotyleans that includes members of the superfamilies Discoceloidea and Leptoplanoidea (figure 3). However, our ancestral state reconstruction was unable to confidently reconstruct development type at deeper nodes in the polyclad tree; i.e. the base of cotylean clade 1 (scaled likelihoods: indirect = 0.865, direct = 0.135), Cotylea (scaled likelihoods: indirect = 0.874, direct = 0.126), Stylochoidea (scaled likelihoods: indirect = 0.547, direct = 0.453), Acotylea (scaled likelihoods: indirect = 0.488, direct = 0.512) and Polycladida (scaled likelihoods: indirect = 0.659, direct = 0.341). The early branching cotylean clade 1 and acotylean Stylochoidea show the greatest variability in mode of development among major polyclad groups. Due to sampling bias in favour of polyclad species, and variation in developmental mode within early diverging polyclad lineages, we were also unable to reconstruct the ancestral mode of development at deeper nodes within Platyhelminthes. To account for this bias, we also reconstructed ancestral mode of development using a previously published phylogeny of flatworms [11]. In this analysis, the ancestor of Polycladida and Prorhynchida Karling, 1974, was reconstructed as a direct developer (scaled likelihoods: indirect = 0.093, direct = 0.908), as were most other ancestral nodes within Platyhelminthes (figure 4). Together, these two ancestral state reconstructions indicate that indirect development may have evolved in the polyclad stem lineage and been lost in the Discoceloidea, Leptoplanoidea and *Theama* (Cotylea clade 1). However, our results could also indicate multiple gains of indirect development in polyclads (i.e. at the ancestral node uniting Euryleptidae, Pseudocerotidae and Prosthiostomidae, and in Planoceridae).

## 3. Discussion

In this study, we have significantly increased the breadth of RNA-Seq sampling in Polycladida to generate a robust phylogenetic hypothesis for this group and provide a framework to investigate the evolution of modes of development in polyclads. We have constructed a large data matrix (greater than 5 million nucleotide positions; 4469 genes) for inferring the phylogeny of Polycladida, which has provided very high confidence in the phylogenetic inferences presented here (BS = 100 within Polycladida and BS > 98 across outgroup branches). This success implies that transcriptomic data will be particularly useful for resolving the phylogeny of Polycladida moving forward.

### 3.1. The phylogeny of Polycladida

Analysis of this large dataset has resulted in a tree that shows some correspondence with single- and few gene-based trees and importantly has resolved uncertainties around early branching lineages in Polycladida (figure 2). Our phylogeny shows full support at all nodes within Polycladida, including deeper nodes that in past studies have often had weak or no support [20,27,28]. We have not modified the nomenclature of clades in Polycladida based on our phylogeny due to the limited taxonomic coverage, but we discuss the implications of the tree topology for systematics and highlight taxa to include in future transcriptome-based analyses. We also discuss limited changes to the classification of some species based on our phylogeny. If not otherwise indicated, our nomenclature and taxonomy definitions follow Dittmann *et al*. [27].

#### 3.1.1. Cotylea

One novel finding resulting from our analysis is a new monophyletic clade (clade 1, figure 2) sister to all other Cotylea that includes the families Cestoplanidae Lang, 1884, Theamatidae, Boniniidae and Diposthidae Woodworth 1898 *sensu* Litvaitis *et al*. [20]. Morphology-based classifications placed Cestoplanidae and Theamatidae within Acotylea [17,18] due to the lack of a ventral cotyl [20]. Recent molecular phylogenies supported Cestoplanidae Lang, 1884 and Theamatidae as early branching members of Cotylea [20,27,28,33]; with Cestoplanidae either as the sister group of all remaining cotyleans [20,27,28,33], or as the sister group of Diposthidae, together forming the sister group of all remaining cotyleans [20,38]. Our analysis places Cestoplanidae within a new clade, with Boninioidea and Diposthidae. In future analyses, the inclusion of other cotyleans identified as early branching (e.g. species belonging to *Anonymus* Lang, 1884, *Chromoplana* Bock, 1922, and *Chromyella* Correa, 1958 [20,27,28]) will be important to see if these species form part of clade 1 and whether clade 1 remains the sister to the rest of Cotylea.

In our reconstruction, *Pseudoceros* Lang 1884 is the sister group of all other pseudocerotids, reflecting topologies of single- and few-gene trees [26–28,33]. The family Pseudocerotidae contains genera with single or duplicated male copulatory organs. Genera with duplicated male organs (e.g. *Thysanozoon* Grube, 1840, *Pseudobiceros* Faubel, 1984) were supported as a monophyletic cluster in the phylogenetic reconstruction of Litvaitis *et al*. [20], although genera with single and duplicated genital organs were intermingled in other studies [20,26–28,33]. Here, *Thysanozoon brocchi* (duplicated male apparatus) is the sister group of ‘*Acanthozoon* or *Thysanozoon* sp.’, where *Acanthozoon* Collingwood, 1876 features a single male apparatus. Although our species determination did not allow distinguishing between *Acanthozoon* and *Thysanozoon*, the hypothesis of a single origin of duplicated male organs put forward by Litvaitis *et al*. [20] suggests the sample labelled ‘*Acanthozoon*/*Thysanozoon* sp.’ in figures 2 and 3 is a *Thysanozoon* rather than an *Acanthozoon*.

Further taxon sampling is required to resolve the relationships within the families Prosthiostomidae and Euryleptidae. Within Prosthiostomidae, *Euprosthiostomum* Bock, 1925 is the sister group of *Enchiridium* Bock, 1913 (this study), although in two recent studies, *Euprosthiostomum* was recovered as the sister group of *Prosthiostomum* Quatrefage, 1845 [20,34]. All three studies used *Euprosthiostomum mortenseni* Marcus, 1948, and recovered the conflicting topologies with high support. Although our tree recovers a monophyletic Euryleptidae consistent with Tsunashima *et al*. [26], other recent phylogenies recovered paraphyletic Euryleptidae [20,27,28,33], which may be attributable to increased taxon coverage, i.e. inclusion of members of Stylostomidae Dittmann *et al*. [28].

#### 3.1.2. Acotylea

The interrelationships of the three acotylean superfamilies recovered here—Stylochoidea as sister to Discoceloidea + Leptoplanoidea—has been proposed by earlier studies [20,26–28,32,33,38]. However, our analysis renders Leptoplanoidea *sensu* Faubel, [17,18] paraphyletic by the inclusion of *Euplana gracilis* within this clade. *E. gracilis* is the first member of Euplanidae to be included in a molecular phylogeny of polyclads. In Faubel's system, Euplanidae belong to the now suppressed superfamily Ilyplanoidea Faubel, 1983 [17,18], and later redefined as Discoceloidea [28]. Here, we transfer Euplanidae to the Leptoplanoidea.

Within Leptoplanoidea, our analysis supports the paraphyly of Gnesiocerotidae Marcus & Marcus, 1966 found in recent studies [30–32,38]. *Gnesioceros sargassicola* (Mertens,1833) is recovered as the sister taxon to all other Leptoplanoidea [20,31,38], but a second species previously assigned to Gnesiocerotidae, *Echinoplana celerrima* Haswell, 1907, forms a derived clade with *Notocomplana* species*.* The sister relationship between *Echinoplana* and *Notocomplana* has also been recovered in Bahia *et al*. [27]. Unlike Notocomplanidae Litvaitis *et al*. [20], which are diagnosed by the absence of a sclerotized stylet on the penis [20], the penis of *Echinoplana* is armed with numerous spines and hooks. Together these data suggest that *Echinoplana* does not belong to Gnesiocerotidae, but may form a new family closely related to Notocomplanidae, or be included in Notocomplanidae (thus prompting a renaming of the family to Echinoplanidae, as *Echinoplana* precedes *Notocomplana*). Including other gnesiocerotids (e.g. *Styloplanocera* Bock, 1913 and *Planctoplanella* Hyman, 1940) in future phylogenomic analyses will determine the valid members of this family.

Our analysis provides a phylogenetic foundation upon which future phylo-transcriptomic and genomic studies may build. The majority of families and genera, erected on a morphological basis by Faubel [17,18] and Prudhoe [16,18], have not been tested here (nor in any one molecular framework) leaving both the monophyly of families, as well as their inclusion in superfamilies, unresolved. We included species from 10 of 28 acotylean families and 5 of 15 cotylean families after Faubel [17,18], and 7 of 18 acotylean families and 5 of 10 cotylean families after Prudhoe [16]. The challenge therefore remains to increase taxonomic sampling in order to establish a new phylogenomics-based system that resolves the conflicting topologies and classifications based on morphology [16–18,39] and limited sampling of genetic markers [20,26–28].

### 3.2. Life-history evolution within Polycladida and Platyhelminthes

Our ancestral state reconstruction highlights the evolutionary complexity of mode of development within Polycladida (figure 3). Our inability to definitively reconstruct character states at the deeper nodes in polyclads is partly due to missing data, but also because early branching clades in both suborders contain taxa with different modes of development.

Although the vast majority of Acotylea studied to date have direct development, larvae are known to occur in two of the three current superfamilies; in Stylochoidea (in the closely related genera *Hoploplana*, *Planocera*, *Stylochus* and *Imogine*, summarized in [14]) and, less frequently, in Leptoplanoidea (*Notoplana australis* (Schmarda, 1859) [39] and possibly *Stylochoplana maculata* Quatrefage, 1845 [40]). An especially interesting case of larval evolution is found in *Planocera*, where *P. reticulata* features a unique eight-lobed, multi-eyed and dorsoventrally flattened Kato's larva but the congener *P. multitentaculata* develops via an eight-lobed, three-eyed, spherical Müller's larva very similar to prosthiostomid, euryleptid and pseudocerotid Müller's larvae [41,42]. Litvaitis *et al*. [20] suggested the presence of larvae could be a synapomorphy for Stylochoidea, but went on to add that because Stylochoidea (*sensu* Poche[43]) has been identified as the most early diverging lineage in Acotylea [20,27,28], it is more likely that larvae are a symplesiomorphy retained from the polyclad ancestor. Our phylogenetic analysis is consistent with Stylochoidea as the sister group of all other acotyleans, and our ancestral state reconstruction does not rule out the possibility that indirect development in Stylochoidea is conserved from an ancestral polyclad. However, descriptions of development are missing in at least one stylochoidean genus in our analysis (*Idioplana*), which may impact our inferences. Furthermore, adding transcriptome and development data from a Latocestidae species, an unrepresented Stylochoidea in this study, may help resolve this node.

Despite most cotylean taxa having indirect development, there was no significant support for this mode of development in the ancestral cotylean. This is likely due to the diversity of development recorded in the early branching clade 1, which may also impact inferences in Acotylea. Within this clade nothing is known about development of the cestoplanids, and the other members exhibit poecilogony (*Pericelis cata,* [22]), indirect (*Boninia divae*, [22]) and direct development (*Theama mediterranea*, [23]). Interestingly though, there are a number of similarities in the development of these three species; the hatchlings have just one eyespot, compared to two or more in other polyclad hatchlings, the egg capsules contain multiple embryos [22,23], and the larvae of *Pericelis cata* and *Boninia divae* have been described as atypical, with a smaller number of lobes that are reduced in size, more similar to the Goette's larvae of acotylean species than the Müller's larvae of other cotyleans [22]. It is possible that these modes of development represent intermediate evolutionary stages between acotylean direct development and the derived cotylean indirect development, or indirect development in both clades.

There are three possible evolutionary scenarios for indirect development in polyclads based on our findings; the first is that a Müller's type larva could be the ancestral polyclad condition, which has been reduced to Goette-like larvae in the stylochids and cotylean clade 1 and lost completely in most Leptoplanoidea + Discoceloidea. The second is that indirect development via a Goette-like larvae (as found in some acotyleans and in some cotylean clade 1 taxa) may be the ancestral condition in polyclads, from which Müller's larvae have independently evolved several times (in *P. reticulata* and in most cotyleans). Third, instead of an ancestral polyclad larva, Müller's and Goette's larvae could be evolutionarily derived larval types within the polyclads that have each evolved several times independently. Although our present analyses cannot shed light on which scenario is more likely, they provide the framework for future analyses and highlight key taxa for further investigations into the mode of development, i.e. *Idioplana atlantica* (Bock, 1913) and Cestoplanidae.

Our second analysis reconstructed the ancestral development type for Platyhelminthes (figure 4) using a previously published phylogeny [11]. This second analysis allowed us to infer the ancestral development mode for Polycladida and its sister lineage, Prorhynchida, because it included a higher number of non-polyclad flatworm taxa and reduced the polyclad-heavy sampling bias of our first analysis, which minimizes the impact of uncertain developmental modes within Polycladida. This analysis points to polyclad larval stages being intercalated into the life cycle of a direct developing prorhynchid/polyclad ancestor along the polyclad stem lineage or within polyclads. Recent phylogenomic studies of flatworms have suggested it is more parsimonious to view polyclad larvae as one or more independent acquisitions limited to this group [10,11], and our analysis supports this.

These interpretations bring into question the hypothesis that indirect development in polyclads is retained from a spiralian ancestor with a biphasic life cycle and trochophore-like larva. This is a long-standing idea based on morphological similarities in larval stages (e.g. larval lobes and ciliary bands) and supported by the presence of spiral cleavage in polyclads and other non-flatworm spiralians [9,41,42], and similar embryonic origins of polyclad and spiralian trochoblasts [44]. Our analyses, instead, suggest that similarities in larval characters of polyclad and trochophore larvae may not be phylogenetically congruent, and may have evolved convergently. Another interpretation is that some elements of indirect development in a spiralian ancestor may have been secondarily derived in polyclads using some combination of novel and conserved developmental pathways. However, to reconstruct the developmental mode of the ancestral flatworm, and inform on homology of polyclad and trochophore larvae, future phylogenetic and ancestral state reconstruction analyses would require the inclusion of many more non-polyclad flatworms and multiple species from sister clades to the flatworms (e.g. nemerteans and annelids [2,45,46]). At present, transcriptomic data for an increasing diversity of these taxa is available [47,48], but data on mode of development for many of these species is lacking.

The evolution of larvae within Spiralia and Metazoa is a convoluted and recurring topic. In order to better describe the origins of indirect development, analyses need to include species that span as much variation in mode of development as possible. In both of our analyses, the equal rates model was preferred (based on AICc scores), implying that the gain or loss of indirect development occurs at similar rates in this group. This may not be the appropriate model of evolution given the complexity of mode of development, but it highlights that a more thorough sampling of this character, and deeper look into its components, may be beneficial for future investigations of indirect development. It might be the inclusion, and the study, of direct developing species that may reveal interesting findings that aid our interpretation of life-history evolution. For example, a vestigial prototroch during embryogenesis in an early branching, direct developing nemertean, for example, suggests that the trochophore larvae were lost in this clade [45].

## 4. Conclusion

This study represents the first phylogenomic framework of polyclads and the first ancestral state reconstructions of mode of development within polyclads and flatworms. Together these analyses revealed the macroevolutionary distribution of developmental modes across superfamilies, families and genera of polyclads, and sheds light on the ancestral condition for many clades. They suggest that indirect development may have evolved secondarily within, or along the lineage leading to, Polycladida and has likely been gained or lost several times. Our findings support the hypothesis that indirect development (and associated characters, such as larval ciliary bands) is homologous among prosthiostomid, euryleptid and pseudocerotid cotyleans, but our analysis was unable to resolve homology of indirect development between the polyclad suborders (Cotylea and Acotylea). Homology of indirect development between flatworm orders (Polycladida and Neodermata), and among flatworms and other spiralian lineages, seems unlikely. Alternative scenarios, such as multiple losses of ancestral flatworm larvae, remain possible but are less parsimonious. Increased taxon sampling with robust transcriptomic (or genomic) data will allow for a more detailed understanding of the complex evolution of different larval forms within Polycladida and across flatworms more generally. Polyclad and neodermatan flatworms make excellent systems for understanding how indirect development evolves and larval characters diversify, particularly regarding the intercalation hypothesis.

## 5. Methods

### 5.1. Organismal sampling

One or more specimens of each of the 21 representative species were collected in intertidal and shallow subtidal, in tide pools or via snorkelling or SCUBA (self-contained underwater breathing apparatus; under AAUS certification) using direct, non-destructive collecting under rocks. *Theama mediterranea* was extracted from sand samples collected near Rovinj, Croatia (see [49]). A visual examination was used for confirmation of identity for 15 species: *Boninia divae* (Hyman, 1955), *Prosthiostomum acroporae, Enchiridium periommatum*, *Idioplana atlantica*, Planoceridae sp., *Phaenocelis* sp. *Phrikoceros mopsus* (Marcus, 1952), *Thysanozoon* or *Acanthozoon* sp., *Pseudobiceros damawan* Newman & Cannon, 1994, *Pseudoceros paralaticlavus* Newman & Cannon, 1994, *Theama mediterranea, Euplana gracilis, Gnesioceros sargassicola, Euprosthiostomum mortensi* and *Thysanozoon brocchi* (Risso, 1881))*.* The identification of the other six species was carried out using morphological analysis of histological sections (see methods below). At least one specimen was placed in RNAlater solution (Qiagen, Hilden, Germany) for RNA preservation and frozen at −80°C within one week of collection to prevent RNA degradation. A second specimen of each species, when available, was fixed as a voucher for morphological analysis, first in 4% formalin using the frozen formalin technique [50] and subsequently preserved in 70% ethanol for long-term storage. For histology, specimens in 70% ethanol were graded into 100% ethanol, cleared in Histoclear (National Diagnostics) for 1 h, infiltrated with 1 : 1 histoclear/paraffin for 24 h and equilibrated in molten paraffin for 24 h (all steps performed at 60°C). Specimens were then embedded in fresh paraffin and left to harden at room temperature for 24 h. Specimens were sectioned in the sagittal plane at 8 µm on a rotary microtome, mounted on glass slides and stained with Masson's trichrome [51]. Identification to genus level was achieved using the taxonomic monographs of Faubel ([17], [18]) and Prudhoe [16]. Species-level ID was achieved by consulting the species descriptions in the literature and also verified by comparing 28S rDNA sequence data from our transcriptomes to the polyclad 28S rDNA sequences available on NCBI Genbank. Specimens not completely used up by RNA extraction were deposited in the Smithsonian National Museum of Natural History (NMNH) and are available for study under the catalogue numbers provided in the electronic supplementary material, table S1.

We generated RNA-Seq data for 21 polyclad species and downloaded data for six additional polyclad species from the NCBI Sequence Read Archive (SRA). We also obtained data from nine outgroup species from the SRA: four species from Prorhynchida (the sister taxon to Polycladida [11], including *Geocentrophora applanata* (Kennel, 1888), *Prorhynchus alpinus* Steinböck, 1924, *Prorhynchus* sp. I Laumer & Giribet, 2014, and *Xenoprorhynchus* sp. I, three species from the sister taxon of Polycladida + Prorhynchida [11], including *Gnosonesimida* sp. IV Laumer & Giribet, 2014, *Schmidtea mediterranea* (Benazzi *et al*., 1975) and *Taenia solium* (Linnaeus, 1758), one species from Macrostomorpha (*Microstomum lineare* (Müller OF, 1773)), and one species from Catenulida (*Stenostomum leucops* (Duges, 1828)). Specimen data and SRA accession numbers are listed in the electronic supplementary material, table S1.

### 5.2. RNA extraction and sequencing

A 20–100 mg tissue sample was taken from the anterior of each animal and homogenized using a motorized pestle. In some cases, the specimen was so small the entire animal was used. For *Theama mediterranea*, 20 adults, starved for 1 month, were extracted in a single tube using a protocol detailed in [10]. For all other polyclads, the tissue was homogenized for 1–2 min, then it was flash-frozen in liquid nitrogen for subsequent homogenizing, until tissue mixture was fully uniform. TriZOL Reagent (Life Technologies, Carlsbad, CA, USA), 500 µl, was then added and the mixture was completely homogenized. Once this process was complete, an additional 500 µl of TriZOL Reagent was added to the solution and the mixture was left at room temperature for five min. Following the 5 min incubation, 100 µl of 1-Bromo-3-chloropropane was added to the solution, which was subsequently mixed thoroughly by vortexing the sample for 10s. The mixture was then left at room temperature for 5 min, and then centrifuged at 16 000 g for 20 min at 8°C. The top aqueous phase was then removed and placed in another tube where 500 µl of 100% isopropanol was added and stored for 1 h at −20°C for RNA precipitation.

After precipitation, the samples were centrifuged at 17 200 g for 10 min at 4°C. The supernatant was then removed, and the pellet was washed with freshly prepared 75% ethanol. The sample was then centrifuged at 7500 g for 5 min at 4°C. The supernatant was removed, and the pellet air-dried for 1 to 2 min (or until it looked slightly gelatinous and translucent). The total RNA was then re-suspended in 10–30 µl of Ambion Storage Solution (Life Technologies, Carlsbad, CA, USA), and 1 µl of SUPERase•In (Thermo Fisher Scientific, Waltham, Massachusetts, USA) was added to prevent degradation.

Total RNA samples were submitted to the DNA Sequencing Facility at University of Maryland Institute for Bioscience and Biotechnology Research, MD, USA or The Hospital for Sick Children Centre for Applied Genomics in Toronto, ON, Canada, where quality assessment, library preparation, and sequencing were performed. RNA quality assessment was done with a Bioanalyzer 2100 (Agilent Technologies, Santa Clara, CA, USA), and samples with a concentration higher than 20 ng µl^−1^ were used for library construction. Library preparation used the Illumina TruSeq RNA Library Preparation Kit v2 (Illumina, San Diego, CA, USA) and 200 bp inserts; 100 bp or 125 bp (*Theama* and *Boninia*), paired-end reads were sequenced with an Illumina HiSeq1000 and HiSeq2000 sequencers (Illumina, San Diego, CA, USA).

### 5.3. Quality control and assembly of reads

Reads that failed to pass the Illumina ‘Chastity’ quality filter were excluded from our analyses. Reads passing the quality filter were assembled using Trinity (version 2.4.0 for most, but version 2.6.6 for species *Boninia divae* and *Theama mediterranea*; [52]) with default settings, which required assembled transcript fragments to be at least 200 bp in length. Reads were trimmed pre-assembly for the species *Boninia divae* and *Theama mediterranea* using Trimmomatic [53]. Assemblies are available at https://doi.org/10.6076/D1JG60. Assembly quality was assessed using BUSCO v5.4.2 [54].

### 5.4. Orthology assignment

Translated transcript fragments were organized into orthologous groups corresponding to a custom platyhelminth-specific core-orthologue set of 9157 protein models (constructed in the same manner as in [55]) using HaMStR (version 13.2.6; [56]), which in turn used FASTA (version 36.3.6; [57]), GeneWise (version 2.2.0; [58]) and HMMER (version 3.1b2; [59]). In the first step of the HaMStR procedure, substrings of assembled transcript fragments (translated nucleotide sequences) that matched one of the platyhelminth protein models were provisionally assigned to that orthologous group. To reduce the number of highly divergent, potentially paralogous sequences returned by this search, we set the E-value cutoff defining an HMM hit to 1 × 10^−5^ (the HaMStR default is 1.0) and retained only the top-scoring quartile of hits. In the second HaMStR step, the provisional hits from the HMM search were compared to our reference taxon, *Echinococcus granulosus* (Batsch, 1786), and retained only if they survived a reciprocal best BLAST hit test with the reference taxon using an E-value cutoff of 1 × 10^−5^ (the HaMStR default was 10.0). In our implementation, we substituted FASTA for BLAST [60] because FASTA programs readily accepted our custom amino acid substitution matrix (POLY90). Both the Platyhelminthes core-orthologue set and custom substitution matrix are available at https://doi.org/10.6076/D1JG60.

The Platyhelminthes core-orthologue set was generated by first downloading all available platyhelminthes clusters with 50% similarity or higher from UniProt [61] (70 698 clusters). Excluding clusters that contained only one sequence left 20 874 clusters. We calculated the sequence similarity of each cluster and as a heuristic, decided to remove clusters whose per cent identity was less than 70%, which left 20 549 clusters. We then assessed the number of times each taxon was represented within those clusters. *Echinococcus granulosus* was identified as the most closely related, most abundant taxon (9157 associated clusters with 70% similarity or higher) and was therefore selected as the reference taxon for the custom HaMStR database. We constructed the platyhelminthes HaMStR database by following the steps given in the HaMStR README file, which included generating profile hidden Markov models for each cluster using HMMER. Our platyhelminthes HaMStR database contained 9157 orthologous groups. All protein sequences for *Echinococcus granulosus* (UniProt/NCBI taxon ID 6210) were downloaded from UniProt and used to generate the BLAST database for HaMStR.

Construction of the custom substitution matrix (PLATY90) followed the procedure outlined in Lemaitre *et al*. [62], which used only greater than 90%-similarity platyhelminthes clusters downloaded from UniProt with singleton clusters removed. The use of a taxonomically focused amino acid substitution matrix follows similar procedures used in arthropods [63] and gastropods [55,64] that seek to improve the amino acid alignments performed in the process of a phylogenomic workflow. In this protocol, a block is defined as a conserved, gap-free region of the alignment. Our blocks output file contained 205 562 blocks.

### 5.5. Construction of data matrix and paralogy filtering

Protein sequences in each orthologous group were aligned using MAFFT (version 7.187; [65]). We used the –auto and –add fragments options of MAFFT to align transcript fragments to the *Echinococcus granulosus* reference sequence, which was considered the existing alignment. We converted the protein alignments to corresponding nucleotide alignments using a custom Perl script. A maximum-likelihood tree was inferred using RAxML-NG (RAxML Next Generation version 0.6.0; [66]) for each orthologous group where at least 75% of the taxa were present (4668 orthologous groups) and was given as input to PhyloTreePruner (version 1.0; [67]). Orthologous groups that showed evidence of out-paralogues for any taxa (2530 orthologous groups out of 4426) were pruned according to the default PhyloTreePruner protocol, which removes all additional sequences outside of a maximally inclusive sub-tree. For orthologous groups containing in-paralogues, multiple sequences were combined into a single consensus sequence for each taxon, and orthologous groups for which fewer than 75% of taxa remained were discarded. This process left 4469 orthologous groups eligible for inclusion in our data matrices. Individual orthologous group alignments were concatenated, and codons not represented by sequence data in at least four taxa were then removed.

### 5.6. Phylogenetic analyses

For phylogenetic analysis, the final nucleotide data matrix from transcriptome data was partitioned by codon position by assigning different model parameters and rates to the three codon positions. We conducted the phylogenetic analysis using RAxML-NG (version 0.6.0; [66]). We used the default settings in RAxML-NG and partitioned our dataset by codon position. Each partition was assigned a general time reversible substitution model (GTR; [68]) with a rate heterogeneity model with a proportion of invariant sites estimated (+I) and the remainder with a gamma distribution (+G; [69]), along with stepwise-addition starting trees. For our analysis, 500 bootstrap replicates were generated, and a best tree search was performed with 20 search replicates. To assess whether heterotachy may be impacting our inferences, we also ran a maximum-likelihood analysis with IQ-TREE v2.2.0 [70] under the GHOST model of evolution [71] with ultrafast bootstrap approximation [72]. Data matrices and phylogenetic analysis outputs are available at https://doi.org/10.6076/D1JG60.

### 5.7. Ancestral state reconstruction

Ancestral states were reconstructed for development type (indirect or direct) for the complete polyclad phylogeny plus outgroups (electronic supplementary material, table S3). We assessed fit for two models using the corrected AIC (AICc), where: (i) all transition rates were equal (ER; same as the symmetrical model in this case); (ii) forward and reverse transitions were different between states (all rates different, ARD). The ER model (AICc = 24.77317) was a slightly better fit to the data than the ARD model (AICc = 25.86218 for development type). In order to more confidently infer ancestral states across Platyhelminthes, we also reconstructed ancestral development type for phylum using a previously published phylogeny [11]. The goal of this reconstruction was to reduce bias caused by the increased sampling of polyclad species compared to other groups in our phylogeny. Tree manipulation was conducted using the APE package [73], and the final ancestral state reconstruction analysis and model testing was completed using the rayDISC function in the corHMM package [74]. The package corHMM fits a hidden rates model that treats rate classes as hidden states in a Markov process, employing a maximum-likelihood approach. When a state is missing for a particular species, RayDISC assigns equal likelihoods to both states (indirect or direct development). In this analysis, the marginal ancestral states are returned, which are given as the proportion of the total likelihood calculated for each state at each node. To test for significance of ancestral state reconstruction, we used proportional likelihood significance tests under the rule of thumb that a log-likelihood difference of 2 or greater represents a significant difference. R-scripts are available at https://github.com/goodgodric28/polycladida_phylogenomics.

## Data Availability

Transcriptomes can be accessed at the SRA at NCBI: SRA accession numbers SRR15530025–SRR15530044 (electronic supplementary material, table S1). Critical files from our phylogenomics pipeline are available on DataDryad (https://doi.org/10.6076/D1JG60 [75]). Scripts and files from our ancestral state reconstruction analyses are available on Github (https://github.com/goodgodric28/polycladida_phylogenomics). The data are provided in the electronic supplementary material [76].
